# First-Principles Study of Bimetallic Pairs Embedded on Graphene Co-Doped with N and O for N_2_ Electroreduction

**DOI:** 10.3390/molecules29040779

**Published:** 2024-02-08

**Authors:** Haozhe Dong, Hao Sun, Guanru Xing, Shize Liu, Xuemei Duan, Jingyao Liu

**Affiliations:** Institute of Theoretical Chemistry, College of Chemistry, Jilin University, Changchun 130023, China; donghz21@mails.jlu.edu.cn (H.D.); haosun22@mails.jlu.edu.cn (H.S.); xinggr21@mails.jlu.edu.cn (G.X.); szliu_0520@163.com (S.L.)

**Keywords:** electrocatalytic nitrogen reduction reaction, dual-atom catalysts, density functional theory

## Abstract

The electrocatalytic nitrogen reduction reaction (NRR) is considered a viable alternative to the Haber–Bosch process for ammonia synthesis, and the design of highly active and selective catalysts is crucial for the industrialization of the NRR. Dual-atom catalysts (DACs) with dual active sites offer flexible active sites and synergistic effects between atoms, providing more possibilities for the tuning of catalytic performance. In this study, we designed 48 graphene-based DACs with N_4_O_2_ coordination (MM′@N_4_O_2_-G) using density functional theory. Through a series of screening strategies, we explored the reaction mechanisms of the NRR for eight catalysts in depth and revealed the “acceptance–donation” mechanism between the active sites and the N_2_ molecules through electronic structure analysis. The study found that the limiting potential of the catalysts exhibited a volcano-shaped relationship with the d-band center of the active sites, indicating that the synergistic effect between the bimetallic components can regulate the d-band center position of the active metal M, thereby controlling the reaction activity. Furthermore, we investigated the selectivity of the eight DACs and identified five potential NRR catalysts. Among them, MoCo@N_4_O_2_-G showed the best NRR performance, with a limiting potential of −0.20 V. This study provides theoretical insights for the design and development of efficient NRR electrocatalysts.

## 1. Introduction

Ammonia (NH_3_) is a key precursor in fertilizer synthesis and a carbon-free energy carrier that possesses advantages such as emission-free combustion, convenient storage, and high energy density. Its role in sustainable development is crucial [1,2,3]. However, current industrial ammonia production heavily relies on the energy-intensive Haber–Bosch process, requiring high temperatures and pressure (300–500 °C, 150–300 atm) for NH_3_ synthesis [4]. This process not only consumes a notable amount of energy (approximately 1–2% of global energy consumption annually), but also releases substantial greenhouse gases. As a result, there is an urgent need to explore sustainable alternatives [5]. Electrocatalytic nitrogen reduction (eNRR) has emerged as a promising alternative method due to its mild reaction conditions, sustainability, and environmental friendliness, potentially replacing the conventional Haber–Bosch process [6]. However, the activation of N_2_ molecules faces challenges due to the high bond energy of the N≡N bond (941 kJ·mol^−1^), low polarizability, and lack of a dipole moment [7]. Consequently, NRR catalysis often requires a high limiting voltage to overcome these obstacles. Additionally, the simultaneous hydrogen evolution reaction (HER) during eNRR compromises the NH_3_ selectivity and Faradaic efficiency (FE) [8]. Thus, the focus lies in developing electrocatalysts with outstanding catalytic activity, high selectivity, and superior FE for large-scale industrial eNRR applications. 

In recent years, atomically dispersed transition metal (TM) catalysts, including single-atom catalysts (SACs) and dual-atom catalysts (DACs) have garnered substantial interest in the field of catalysis due to their high atomic utilization, tunable electronic structures, and unique local chemical environments [9,10,11,12]. Currently, there have been a large number of computational and experimental studies on SACs for eNRR. In atomically dispersed transition metal catalysts, choosing suitable support materials is crucial to prevent metal agglomeration and enhance the catalyst stability [13,14,15,16]. Two-dimensional materials, such as graphene, C_2_N, g-C_3_N_4_, h-BN, and MXene, have been commonly used as substrates for SACs or DACs owing to their large surface area, ordered structure, and controllable electronic properties. Among these, graphene stands out as an ideal substrate for SACs and DACs due to its high charge carrier mobility, excellent conductivity, and chemical stability [17,18,19,20,21]. Recently, special attention has been directed towards the family of transition metal atoms anchored on N-doped graphene (TM-N_x_/G), such as TM-N_4_ [22], due to its high reactivity and stability. However, they often exhibit lower overpotential in the hydrogen evolution reaction (HER), leading to lower Faradaic efficiency for the NRR. To address this issue, strategies involving the adjustment of active sites and/or coordination environments have been proposed to improve the catalytic performance and stability [23,24,25].

As an extension of SACs, DACs utilize the synergistic effect introduced by the second metal to maintain the low oxidation state of transition metals and effectively activate inert molecules [26]. Experimental and theoretical evidence indicates that some DACs, such as Mn_2_@C_2_N [27], FeM-N_6_-C [28]; Fe_2_N_4_@graphene [29], FeRu@N_4_-P [30], and Mo_2_@PC_6_ [31], exhibit superior catalytic performance in eNRR compared to Ru (0001) (−0.98 V) [32]. Nevertheless, the stability and catalytic performance of these catalysts are highly sensitive to the coordination environment of the metal centers [33]. Previous studies have demonstrated that adjusting the atoms in the first coordination layer around the transition metal atoms is an effective strategy to modulate the catalytic performance. In TM-N_x_/G, the introduction of additional non-metallic dopants (such as oxygen, boron, sulfur, etc.) in the first coordination layer around the transition metal atom significantly enhances the catalytic performance beyond N atom coordination alone [34,35,36]. For instance, in the M_2_N_6_/G system, the Mn_2_ diatomic catalyst with O and N co-coordination (Mn_2_ON_5_/G^α^) demonstrates significantly improved NRR activity and selectivity [37]. In addition, by employing different transition metals and ligands, DACs have the potential to break linear proportionality in certain reactions [38]. DACs exhibit promising prospects in eNRR applications due to the ability to flexibly choose different metal combinations and coordination environments, and the number of active centers.

A recent experimental approach demonstrated a synthetic method to produce a library of DACs using macrocyclic precursors through an encapsulation–thermal decomposition synthesis strategy. This led to the successful synthesis of a series of nitrogen and oxygen co-doped porous carbon-supported DACs (MM′@N_4_O_2_-G) [39]. These complexes allow for a wide range of metal center modulation, including 3d transition metals (Mn, Fe, Co, Ni, Cu, Zn) and noble metals (Pd, Rh, Pt). Furthermore, by independently controlling elements, the formation of both homonuclear and heteronuclear bimetallic centers, such as Fe_2_, Co_2_, FeCu, and CuCo, can be achieved. The study revealed that these DACs exhibit excellent catalytic activity and stability in the oxygen reduction reaction (ORR), particularly with FeCu-DAC outperforming corresponding SACs and other Fe-based DACs. This progress has inspired us to design dual-atom catalysts with diverse metal combinations and N_4_O_2_ coordination for the NRR. 

In this study, we employed density functional theory (DFT) to investigate the catalytic performance of a series of DACs with the M_2_N_4_O_2_ motif embedded in graphene (MM′@N_4_O_2_-G) for the NRR to NH_3_. In terms of metal selection, considering the common metals found in the active centers of nitrogenase (such as Mo, V, and Fe) [36], we chose these three metals as M, while M′ included 3d transition metals (Sc~Zn), 4d transition metals (Zr, Nb, and Mo), and 5d transition metals (Hf, Ta, W, and Re). The computational results indicate that these five DACs exhibit good catalytic performance for the NRR while inhibiting the side reaction (HER). Detailed analysis of the electronic structure properties of these catalysts revealed that the high activity of the DACs stemmed from the effective modulation of the intermediate stability and synergistic effects between the active sites. This study is anticipated to offer an important reference and guidance for the development of highly active and selective DACs.

## 2. Results and Discussion

### 2.1. Catalyst Structure and Stability

**Figure 1:**
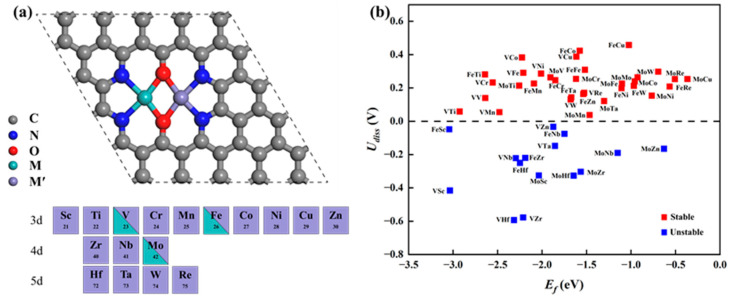
(**a**) Schematic of the optimized catalyst structure. (**b**) Corresponding formation energy and dissolution potential of the catalyst.

Based on the successful synthesis of DACs possessing N_4_O_2_ coordination in experiments, we constructed 48 types of MM′@N_4_O_2_-G DACs on this substrate, including 3 homonuclear DACs, VV/MoMo/FeFe@N_4_O_2_-G, and 45 heteronuclear DACs, MM′@N_4_O_2_-G, where M = V/Fe/Mo and M′ = Sc ~ Zn, Zr, Nb, Mo, Hf, Ta, W, and Re. The optimized structures are shown in Figure 1a. To assess the thermodynamic stability of these catalysts, we calculated the formation energy (*E_f_*) as follows:

$$E_{f}=(E_{cat}-E_{sub}-E_{M}-E_{M^{\prime }})$$

where *E_cat_* is the total energy of the optimized catalyst, *E_sub_* is the energy of the catalyst substrate, and *E_M_* is the energy of an individual metal atom in its most stable bulk phase structure. Subsequently, we calculated the dissolution potential (*U_diss_*) to evaluate the electrochemical stability:

$$U_{diss}=U_{diss}^{0}-E_{f}/N_{e}$$

where *U_diss_* represents the standard dissolution potential of the bulk metal, denoted as $U_{diss}^{0}=\left[U_{diss}^{0}\left(M\right)+U_{diss}^{0}\left(M^{\prime }\right)\right]/2$ for the diatomic system; *N_e_* represents the number of electrons transferred during metal dissolution, expressed as *N_e_* = [*N_e_*(*M*)+ *N_e_*(*M′*)]/2 [28]. According to the calculated results shown in Figure 1b, the *E_f_* values of these catalysts are all negative, indicating their thermodynamic stability. Moreover, among the 48 diatomic catalysts, 33 exhibit *U_diss_* values greater than 0, indicating good electrochemical stability while maintaining thermodynamic stability. Consequently, they are potential candidate catalysts for further investigation. Additionally, Bader charge analysis was used to calculate the charge transfer between the anchored metal atoms and the substrate. As shown in Table S1, a substantial number of electrons transfer from the anchored metal atoms to N_4_O_2_-G, indicating a strong interaction between the metal and the substrate.

### 2.2. Performance of MM′@N_4_O_2_-G for Electrocatalytic NRR

#### 2.2.1. Screening of Catalysts

**Figure 2:**
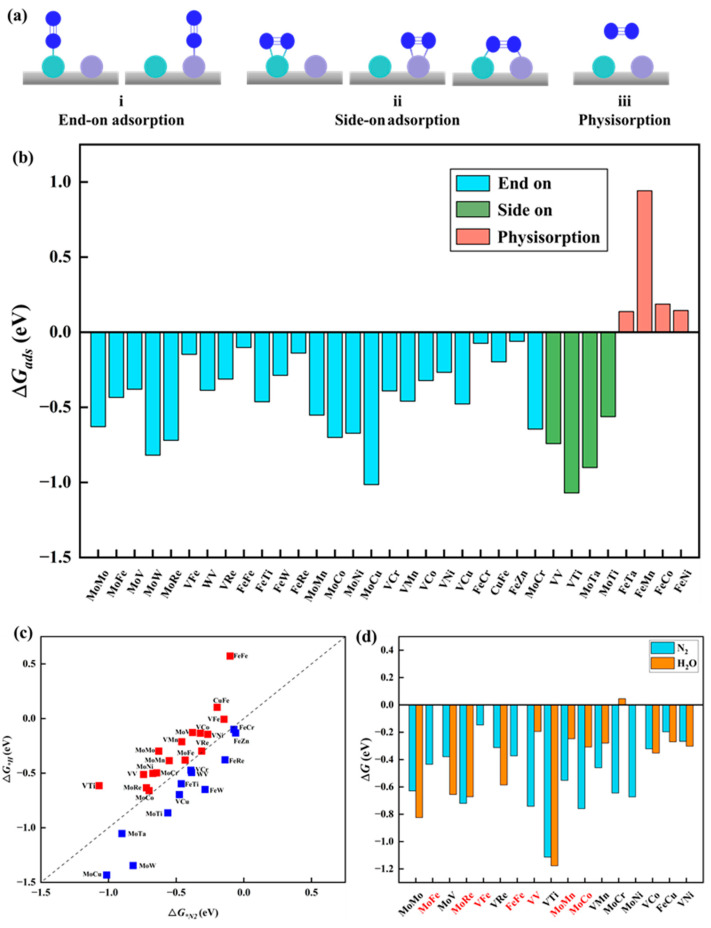
(**a**) Schematic structure of N_2_ adsorption on catalyst. (**b**) The adsorption free energy of N_2_ on the catalyst. (**c**) Comparison between ΔG_*H_ and ΔG_*N__2_. (**d**) Comparison between ΔG_*N__2_ and ΔG_*H__2*O*_, where the red coordinates represent the catalysts that meet the requirements.

N_2_ adsorption. The adsorption and activation of N_2_ molecules represent the initial and crucial steps in the NRR process. To determine the most favorable adsorption structures, three types of adsorption configurations were considered for homonuclear DACs: one involving end-on adsorption, where one N atom of N_2_ forms a bond with a single metal atom, and two types of side-on adsorption, one with two N atoms of N_2_ adsorbed on a single metal atom and the other with two N atoms of N_2_ bonded to two different metal atoms. Regarding heteronuclear DACs, given the diverse nature of the two metal active sites, five adsorption configurations were considered, comprising two end-on adsorptions and three side-on adsorptions, as shown in Figure 2a. Figure 2b presents the adsorption free energy (ΔG_*N2_) of the optimized N_2_ adsorption configurations on 33 catalysts. Notably, except for the physical adsorption of N_2_ molecules on four catalysts (MM′@N_4_O_2_-G, MM′ = FeTa, FeMn, FeCo, and FeNi), the other 29 catalysts demonstrate the chemical adsorption of N_2_ molecules on the surface, with adsorption free energy values ranging from −0.06 to −1.07 eV. Among them, N_2_ molecules exhibit side-on adsorption on four catalysts (MM′@N_4_O_2_-G, MM′ = VV, VTi, MoTa, and MoTi), while end-on adsorption is mainly observed on 25 other catalysts, mainly at the M or M′ active sites of MM′@N_4_O_2_-G. Thus, based on the calculated adsorption free energy of N_2_, only four catalysts (MM′@N_4_O_2_-G, MM′ = FeTa, FeMn, FeCo, and FeNi) are excluded.

**Figure 3:**
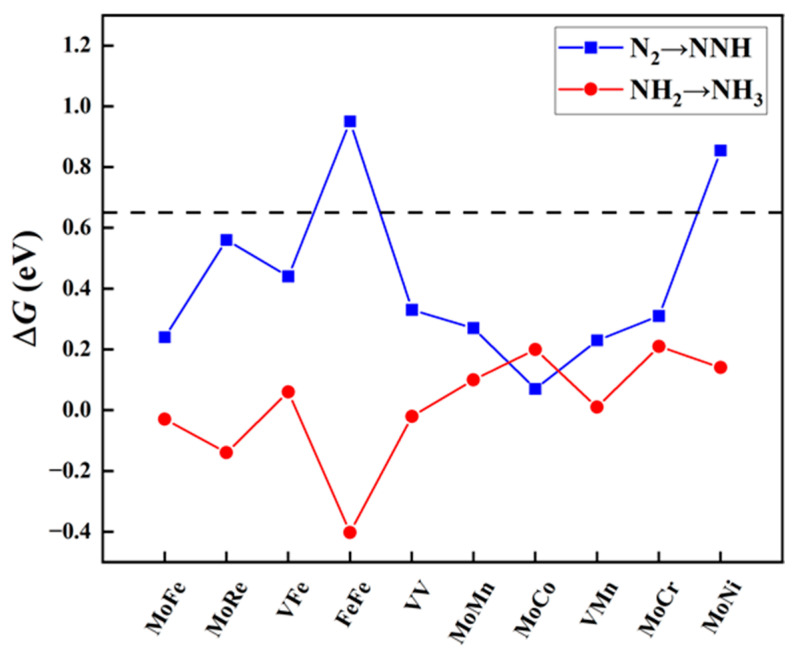
Free energy changes (Δ*G_N_*_2→*NNH*_ and Δ*G_NH_*_2→*NH*3_, eV) for the first and last protonation steps on MM′@N_4_O_2_-G. The black dashed line indicates the screening criteria (∆*G* = 0.65 eV).

Competitive adsorption at active sites. In the NRR, competitive adsorption among different species at active sites is an important concern, especially under experimental conditions where hydrogen protons and water molecules in the solution may compete for adsorption with N_2_ molecules at the active sites on the catalyst surface. If the catalyst exhibits stronger adsorption of hydrogen atoms compared to N_2_ molecules, this could potentially lead to the occupation of active sites by hydrogen atoms, favoring the promotion of the HER over the NRR. Such a case leads to the decreased Faradaic efficiency of the NRR, thereby impacting the selectivity and efficiency of the overall reaction. To evaluate this, we compared the adsorption free energies of *H (ΔG_*H_) and ΔG_*N2_. Figure 2c illustrates that 17 catalysts exhibit stronger adsorption towards N_2_ molecules than towards H atoms, indicating that these catalysts are more conducive to facilitating an efficient electrochemical NRR. Additionally, if solvent molecules cover metal atoms instead of N_2_ molecules, it can affect the sustained NRR and lead to the oxidation of transition metals in the aqueous electrolyte, ultimately hindering the progress of the reaction [40]. To address this issue, the adsorption free energy of H_2_O molecules (ΔG_*H__2*O*_) on the remaining 17 catalysts was calculated, as shown in Figure 3d. Ten MM′@N_4_O_2_-G catalysts (MM′ = MoFe, MoRe, VFe, FeFe, VV, MoMn, MoCo, VMn, MoCr, and MoNi) were identified for their favorable adsorption behavior towards N_2_ molecules within the competitive adsorption environment. These catalysts show promising potential for an efficient NRR.

The protonation of the first step and the last step. The eNRR involves six proton-coupled electron transfer (PCET) steps. Despite the specific mechanism of the NRR, the hydrogenation reactions of the first step (*N_2_ + H^+^ + e^−^ → *NNH) and the sixth step (*NH_2_ + H^+^ + e^−^ → *NH_3_) are the most common elementary steps. Previous studies have indicated [41,42,43] that these two steps usually act as the potential determining steps (PDS) in the NRR, demonstrating the largest free energy change throughout the reaction process. To evaluate these crucial steps, we calculated the reaction free energy change for the protonation of the first step (Δ*G_N_*_2→*NNH*_) and the last step (Δ*G_NH_*_2→*NH*3_). Using a standard value of 0.65 eV, we performed preliminary screening for the aforementioned ten candidate diatomic catalysts. As shown in Figure 3, eight catalysts met the criteria set in this study, including one homonuclear DAC (VV@N_4_O_2_-G) and seven heteronuclear DACs (MM′@N_4_O_2_-G, MM′ = MoCo, MoFe, MoCr, MoMn, MoRe, VFe, and VMn). Additionally, it is notable that, for most diatomic catalysts, the free energy change for the protonation of N_2_ to form NNH in the first step is greater, except for MoCo@N_4_O_2_-G, where the Δ*G_NH_*_2→*NH*3_ (0.20 eV) is greater compared to Δ*G_N_*_2→*NNH*_ (0.07 eV).

**Figure 4:**
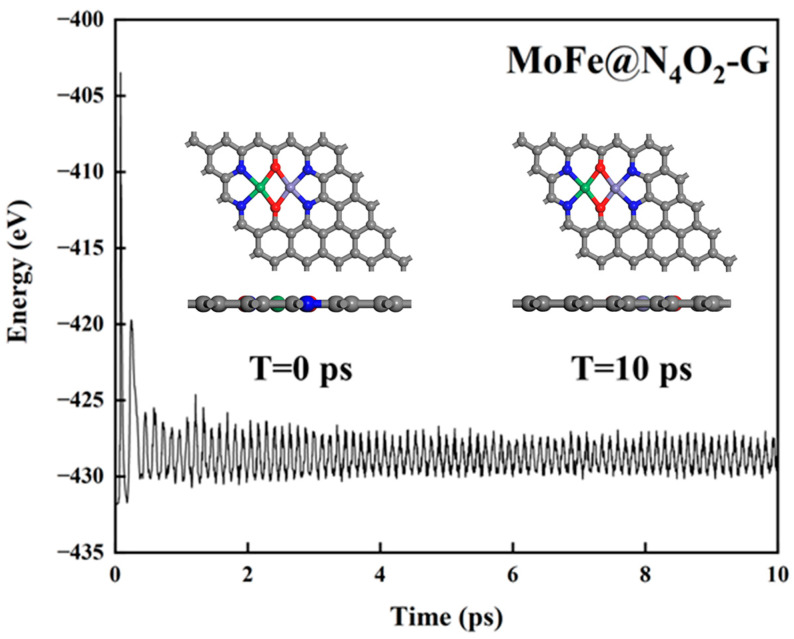
Total energy variation of MoFe@N_4_O_2_-G for AIMD simulation at 500 K for 10 ps. The C, N, O, Mo, and Fe atoms are labeled as gray, blue, red, green, and lavender balls, respectively.

Thermal stability of the catalyst. The stability of catalysts under operating conditions is crucial for their practical application. To further assess the thermal stability of the 8 catalysts, we conducted a 10 ps ab initio molecular dynamics (AIMD) simulation at a temperature of 500 K. Figure 4 and Figure S1 illustrate the variations in temperature and energy over time for these catalysts. Clearly, during the 10 ps duration, there were negligible structural changes observed in the catalysts, indicating excellent thermal stability.

#### 2.2.2. NRR Reaction Mechanism

**Figure 5:**
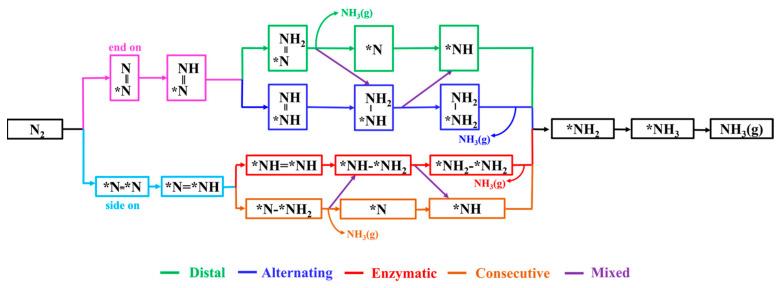
Diagram of possible reaction mechanisms for NRR.

For the eight DACs selected through the aforementioned screening process, we conducted detailed calculations of the possible NRR pathways to assess their catalytic performance. Based on the N_2_ adsorption configurations and different hydrogenation sequences of the two N atoms, the electrocatalytic NRR on DAC surfaces typically involves various pathways, as shown in Figure 5. For N_2_ adsorption through the end-on pattern, the NRR can proceed via either distal or alternating pathways for protonation reactions. In the distal pathway, the proton–electron pairs initially react with the N atom away from the adsorption site, resulting in the formation of the first NH_3_. Subsequently, consecutive protonation steps lead to the formation of the second NH_3_. In the alternating pathway, the proton–electron pairs alternately attack the two N atoms, eventually leading to the sequential production of two NH_3_ molecules. Regarding the side-on adsorption pattern, the NRR occurs via two pathways: the enzymatic (red line) and the consecutive pathways (brown line). Additionally, the NRR can also take place through a mixed pathway, alternating between the distal and alternating pathways or between the enzymatic and consecutive pathways.

**Figure 6:**
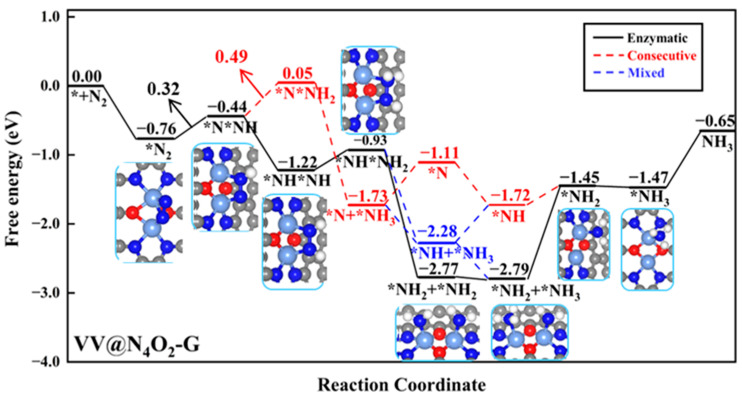
Gibbs free energy diagrams of NRR on VV@N_4_O_2_-G. The C, N, O, H, and V atoms are labeled as gray, blue, red, white, and light blue balls, respectively.

VV@N_4_O_2_-G was the only homonuclear DAC that remained after the screening process. N_2_ molecules exhibit side-on adsorption on the catalyst surface, where two N atoms bond to two V atoms, with ΔG_*N__2_ of −0.76 eV. The Gibbs free energy diagram for the NRR on VV@N_4_O_2_-G and corresponding intermediate structures are shown in Figure 6. As shown in the figure, for the VV@N_4_O_2_-G catalyst, the potential-determining step (PDS) in the consecutive pathway is the second step of the protonation reaction (*NNH + H^+^ + e^−^ → *NNH_2_), with a Δ*G* value of 0.49 eV. The PDS of both the enzymatic and mixed pathways is the first protonation step (*N_2_ + H^+^ + e^−^ → *NNH), with a ΔG value of 0.32 eV. The first four protonation steps in the two pathways lead to the *NHNH_2_ intermediate. In the fifth protonation step, a proton–electron pair attacks one N atom in the *NHNH_2_ intermediate, forming *NH_2_NH_2_ or *NHNH_3_, with ΔG values of 1.84 and −1.35 eV, respectively, indicating that the former is more feasible in thermodynamical terms. Subsequently, *NH_2_ + *NH_2_ undergoes two hydrogenation steps to produce two adsorbed NH_3_ molecules, with Δ*G* values of 0.02 and −0.02 eV. It is noteworthy that the desorption of the two NH_3_ molecules from VV@N_4_O_2_-G requires relatively high energies, at 1.34 eV and 0.82 eV, respectively. However, previous studies have demonstrated that NH_3_ generated in strong acid solutions can be easily reduced to NH_4_^+^ [44]; hence, the desorption of NH_3_ is not extensively considered here. Our calculations show that for VV@N_4_O_2_-G, the most probable reaction pathway is the enzymatic pathway, with a *U_L_* of −0.32 V.

**Figure 7:**
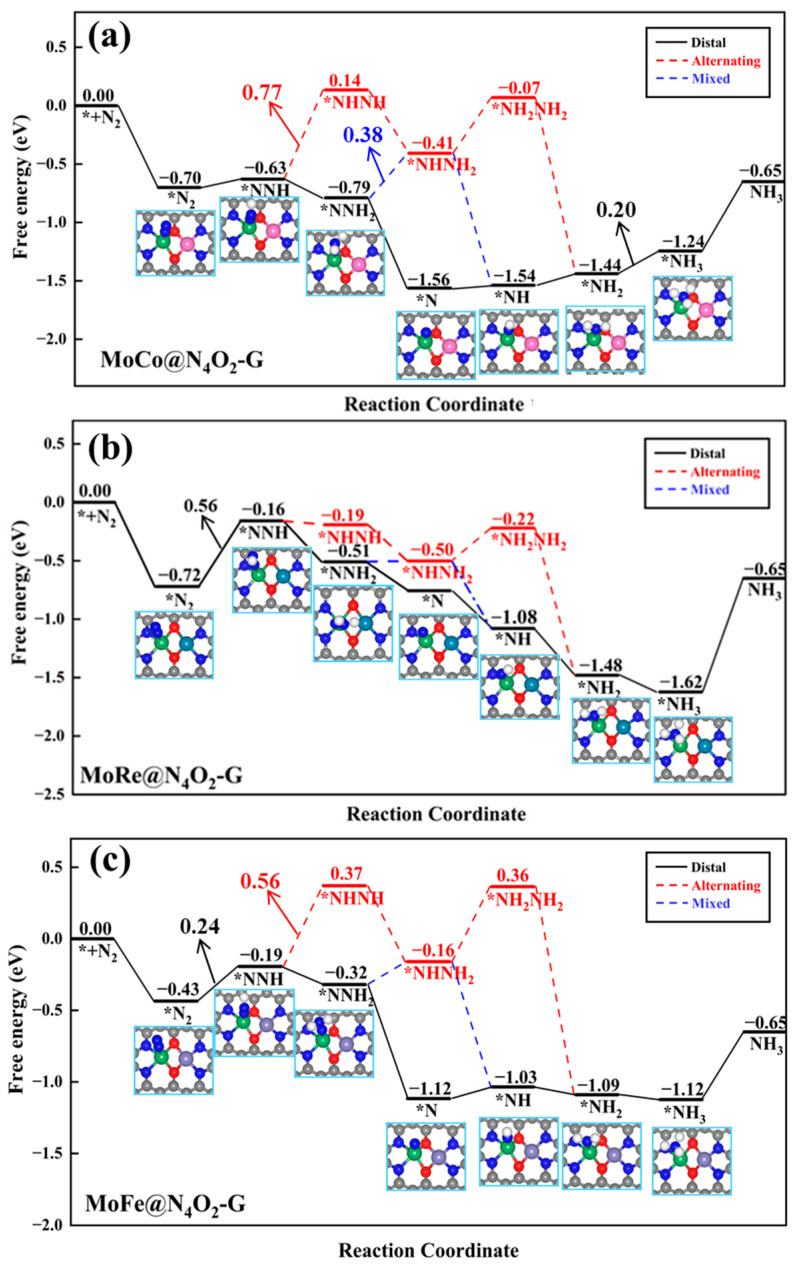
Gibbs free energy diagrams of NRR on (**a**) MoCo@N_4_O_2_-G, (**b**) MoRe@N_4_O_2_-G, and (**c**) MoFe@N_4_O_2_-G. The C, N, O, H, Mo, Co, Re, and Fe atoms are labeled as gray, blue, red, white, green, pink, dark green, and lavender balls, respectively.

In the other seven heteronuclear DACs (MM′@N_4_O_2_-G, MM′ = MoCo, MoCr, MoFe, MoMn, MoRe, VFe, and VMn), N_2_ is adsorbed in an end-on configuration on Mo or V atoms. As depicted in Figure 7a, for the MoCo@N_4_O_2_-G catalyst, the protonation reactions follow three different reaction pathways (distal, alternating, and mixed pathways) with the PDS as the sixth step (*NH_2_ + H^+^ + e^−^ → *NH_3_), the second step (*NNH + H^+^ + e^−^ → *NNH_2_), and the third step (*NNH_2_ + H^+^ + e^−^ → *NHNH_2_), with ΔG values of 0.20 eV, 0.77 eV, and 0.38 eV, respectively. Consequently, the distal pathway becomes the most favorable reaction pathway for MoCo@N_4_O_2_-G. For MoRe@N_4_O_2_-G and VFe@N_4_O_2_-G, as shown in Figure 7b and Figure S2, the PDS for all three pathways is the first protonation step, with Δ*G_max_* values of 0.56 and 0.44 eV, respectively. However, compared to the alternating and mixed pathways, the distal pathway displays superior thermodynamic advantages on these two catalysts. Therefore, the NRR on MoRe@N_4_O_2_-G and VFe@N_4_O_2_-G tends to proceed along the distal pathway. As for the remaining four DACs (MoFe@N_4_O_2_-G, MoCr@N_4_O_2_-G, MoMn@N_4_O_2_-G, and VMn@N_4_O_2_-G), as depicted in Figure 7c and Figure S3, all show a preference for the distal pathway in the NRR. The PDS is represented by *NNH + H^+^ + e^−^ → *NNH_2_, with corresponding *U_L_* values of −0.24, −0.25, −0.27, and −0.23 eV, respectively.

Due to the end-on adsorption of N_2_ on heteronuclear DACs, when a N_2_ molecule adsorbs on one metal atom and undergoes the NRR through a distal pathway, the other metal atom can also serve as a reactive site. We further investigated the NRR mechanism when two N_2_ molecules were simultaneously adsorbed on these seven heteronuclear DACs. Firstly, we studied the co-adsorption of two N_2_ molecules on the diatomic sites. It was found that the second N_2_ molecule only physisorbed on the catalyst surface in the cases of MoCr@N_4_O_2_-G, VMn@N_4_O_2_-G, VFe@N_4_O_2_-G, and MoMn@N_4_O_2_-G, while the adsorption free energies of the second N_2_ molecule on MoFe@N_4_O_2_-G, MoRe@N_4_O_2_-G, and MoCo@N_4_O_2_-G were −0.34, −0.69, and −0.41 eV, respectively. Subsequently, we calculated the free energy changes from *N_2_ + *N_2_ to *NNH + *N_2_ on the latter three catalysts. On MoFe, MoRe, and MoCo, the free energy changes from *N_2_ + *N_2_ to *NNH(Mo) + *N_2_ are 0.32, 0.35, and 0.32 eV, respectively, while the free energy changes from *N_2_ + *N_2_ to *N_2_ + *NNH(M′) are 1.18, 1.76, and 0.62 eV, respectively. The results indicate that on the surfaces of MoFe@N_4_O_2_-G and MoCo@N_4_O_2_-G, the ΔG of this step is larger than the rate-determining step’s free energy obtained when a single N_2_ molecule is adsorbed, indicating that simultaneously adsorbing two N_2_ molecules is not feasible on these two surfaces (Table S2). Conversely, on the MoRe@N_4_O_2_-G surface, the ΔG of this step is lower than the Δ*G_max_* when a single N_2_ is adsorbed, suggesting that the MoRe@N_4_O_2_-G catalyst can simultaneously adsorb two N_2_ molecules for the NRR, preferentially inducing the first hydrogenation of the N_2_ adsorbed on the Mo atom.

**Figure 8:**
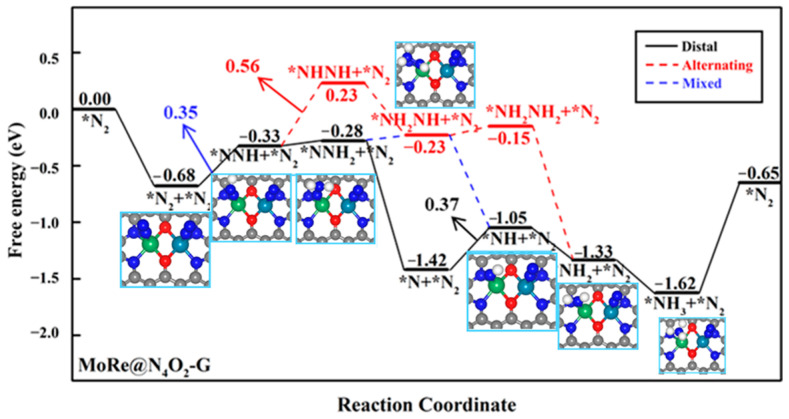
Gibbs free energy diagram of NRR on MoRe@N_4_O_2_-G adsorption of two N_2_ molecules. The C, N, O, H, Mo, and Re atoms are labeled as gray, blue, red, white, green, and dark green balls, respectively.

In the subsequent reaction processes, because both N_2_ molecules have the potential for hydrogenation, we compared the free energy changes of two hydrogenation elementary steps starting from the intermediate *NNH: *NNH + *N_2_ → *NNH + *NNH and *NNH + *N_2_ → *NNH_2_ + *N_2_. The former displays a much higher free energy change (1.11 eV) compared to the latter (0.05 eV). Similarly, for the two elementary steps starting from the intermediate *NNH_2_, the ΔG of *NNH_2_ + *N_2_ → *NNH_2_ + *NNH (1.14 eV) is much higher that of *NNH + *N_2_ → *NNH_2_ + *N_2_. Therefore, we infer that on MoRe@N_4_O_2_-G, the NRR continuously hydrogenates one N_2_ molecule while suppressing the hydrogenation of another N_2_ molecule. The corresponding reaction free energy diagram and optimized intermediate structures are depicted in Figure 8. Computational results indicate that the mixed pathway is the most feasible route and the PDS remains as *N + *N_2_ + H^+^ + e^−^ → *NNH + *N_2_, with a *U_L_* of −0.35 V, significantly lower than the *U_L_* (−0.56 V) corresponding to the case when a single N_2_ adsorbs on the surface. Consequently, it can be inferred that on MoRe@N_4_O_2_-G, the NRR is more inclined towards the adsorbing two N_2_ molecules and follows a mixed mechanism.

### 2.3. Origin of NRR Catalytic Activity

**Figure 9:**
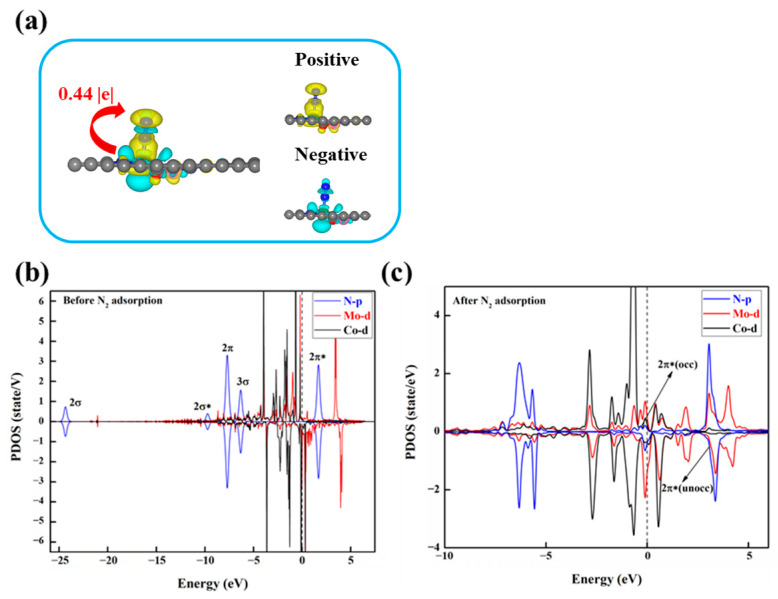
(**a**) Charge density differences after N_2_ adsorption on MoCo@N_4_O_2_-G and Bader charge (Q_*N2_, |e|) after N_2_ adsorption. The C, N, O, Mo, and Co atoms are labeled as gray, blue, red, green, and pink balls, respectively. The electrons accumulation and loss are represented by yellow and cyan areas. (**b**) PDOS before and (**c**) after N_2_ adsorption on MoCo@N_4_O_2_-G. The black dashed line represents the Fermi energy level.

To investigate the underlying factors influencing the activity of DACs in the NRR, we conducted electronic structure calculations on these eight DACs. Firstly, we analyzed the charge transfer between the N_2_ molecule and the catalyst through charge density difference (CDD) and Bader charge analysis. Taking MoCo@N_4_O_2_ as an example, as shown in Figure 9a, evident charge transfer between the active site and N_2_ is observed, with a tendency for charge accumulation near the proximal N atom of N_2_, reducing the charge density between the two N atoms, thereby weakening the chemical bond and facilitating N_2_ activation. Additionally, the increased N-N bond length after N_2_ adsorption also reflects the activation of N_2_. Compared to free N_2_ molecules (d_N-N_ = 1.114 Å), the adsorbed N_2_ exhibits a significantly increased N-N bond length, ranging from 1.148 Å to 1.269 Å, indicating that the electron transfer between the catalyst and N_2_ effectively activates the N_2_ molecule.

In order to gain deeper insights into the fundamental electron transfer mechanism during N_2_ activation, using MoCo@N_4_O_2_-G as an example, the partial density of states (PDOS) of the DACs before and after N_2_ adsorption was studied, as shown in Figure 9b,c. Compared to the free N_2_ molecular orbitals, the 2π and 3σ orbitals of the adsorbed N_2_ shift upwards and exhibit significant hybridization with the Mo 3d orbitals below the Fermi level. This indicates that the unoccupied 3d orbitals of the Mo atom accept electrons from the 2π and 3σ orbitals of the N_2_ molecule, forming bonding states that promote nitrogen adsorption. On the other hand, the unoccupied 2π* orbitals of N_2_ move towards the Fermi level after adsorption, forming partially occupied 2π* orbitals. This suggests that the occupied 3d orbitals of the Mo atom donate electrons to the antibonding orbitals of N_2_, thereby weakening the strength of the N-N bond and facilitating subsequent hydrogenation reactions. Similar situations are observed in the PDOS of other DACs (Figure S4), indicating that N_2_ activation on these catalysts follows an “acceptance–donation” mechanism.

**Figure 10:**
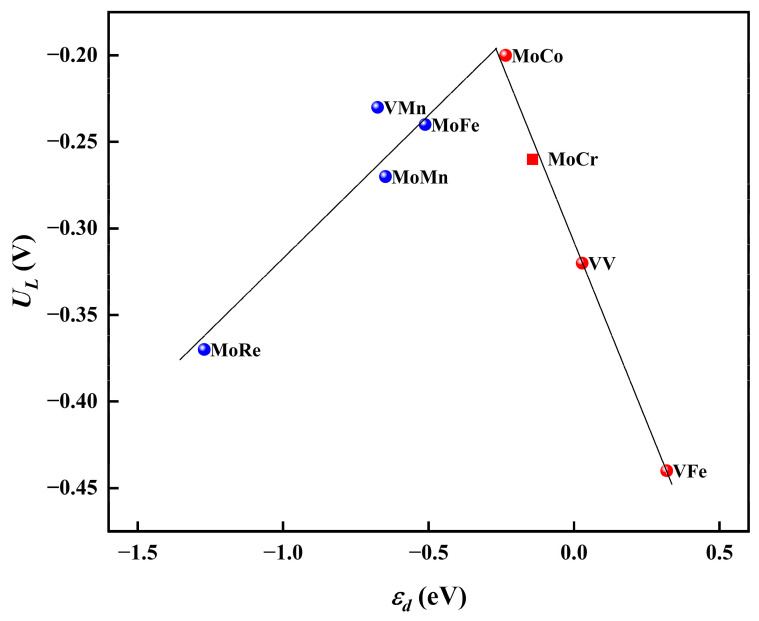
Relationship between *U_L_* and *ɛ_d_* on MM′@N_4_O_2_-G.

Moreover, to investigate the mechanism of the synergistic effects between diatomic sites, we explored the influence of the d-band center (*ɛ_d_*) of the active sites on the reaction activity. Specifically, we evaluated the d-band center of the Mo atom in the case of MoCo@N_4_O_2_-G and assessed the d-band center of the two V atoms in VV@N_4_O_2_-G. As shown in Figure 10, a distinct volcano-shaped relationship exists between the limiting potentials of these eight DACs and the d-band centers of the active metal atoms. Notably, the highly efficient MoCo@N_4_O_2_-G is located near the peak of the volcano plot. The volcano curve suggests that the superior NRR performance of DACs is attributed to the appropriate position of the d-band center. Additionally, it is evident that although the metal M′ (in the case of heteronuclear DAC) does not directly participate in the hydrogenation process of N_2_, the synergistic effects between the M′ and M sites effectively regulate the position of the d-band center of the active site, thereby impacting the reaction activity.

### 2.4. NRR Selectivity of the MM′@N_4_O_2_-G Catalysts

**Figure 11:**
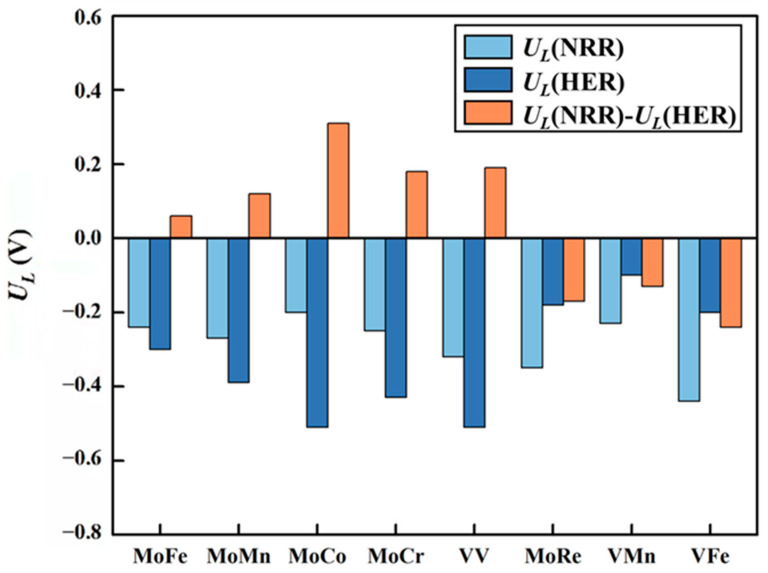
Difference between the limiting potential of NRR (*U_L_*(NRR)) and the limiting potential of HER (*U_L_*(HER)) on the 8 DACs (*U_L_*(NRR)−*U_L_*(HER)).

The main competing reaction during the NRR is the HER. This competition greatly influences the selectivity of the catalyst. The difference between *U_L_*(NRR) and *U_L_*(HER) is commonly used to assess the selectivity of a catalyst. A positive value of *U_L_*(NRR)−*U_L_*(HER) indicates that the catalyst favors the NRR over the HER, while a negative value indicates the opposite. The results of *U_L_*(NRR)−*U_L_*(HER) of eight DACs are shown in Figure 11. It can be seen that MoRe@N_4_O_2_-G, VMn@N_4_O_2_-G, and VFe@N_4_O_2_-G exhibit negative *U_L_*(NRR)−*U_L_*(HER) values, indicating poor selectivity for the NRR. Conversely, the remaining five catalysts (MM′@N_4_O_2_-G, MM′ = MoFe, MoMn, MoCo, MoCr, and VV) all have positive values for *U_L_*(NRR)−*U_L_*(HER). Notably, because four (MM′@N_4_O_2_-G, MM′ = MoFe, MoMn, MoCo, and MoCr) of these five catalysts adsorb N_2_ onto their surfaces via an end-on mode, the initial hydrogenation reaction between *N_2_ and H^+^ can either generate the *NNH intermediate as discussed above or form the *N_2_ + *H intermediate through H^+^ directly adsorbing onto another active site. To determine the more feasible intermediate, we compared the free energy changes of these two intermediates formed on these four catalysts (Table S3). The results show that all four catalysts are more likely to form *NNH. Therefore, these five catalysts (MM′@N_4_O_2_-G, MM′ = MoFe, MoMn, MoCo, MoCr, and VV) exhibit good selectivity and hold potential as catalysts for the NRR.

MoCo@N_4_O_2_-G exhibits the most favorable catalytic performance among the five DACs, with a limiting potential of −0.20 V. Its catalytic activity surpasses that of several other catalysts, including TiV-CG (−0.30 V) [23], FeMo-N_6_-C (−0.63 V) [28], Fe_2_N_4_@graphene (−0.32 V) [29], and Mn_2_ON_5_/G^α^ (−0.27 V) [37].

## 3. Computational Methods

All computations in this study, based on spin-polarized density functional theory (DFT) [45,46], were conducted using the Vienna Ab Initio Simulation Package (VASP 5.4.4) [47,48]. The projector augmented wave (PAW) [49] method was employed to deal with the ion–electron interactions. The cutoff energy for the plane-wave basis set was set to 450 eV. The Perdew–Burke–Ernzerhof (PBE) functional [50] within the general gradient approximation (GGA) was used to describe the electronic exchange-correlation interactions. A (5 × 5) graphene supercell was adopted as the catalyst substrate, with a vacuum layer of 20 Å introduced along the z-axis to eliminate the interaction between periodic images. For structure optimization and electronic structure calculations, Monkhorst–Pack k-point grids of 3 × 3 × 1 and 11 × 11 × 1 were utilized to sample the Brillouin zone. The convergence criteria for energy and forces were set to 10^−5^ eV and 0.02 eV/Å, respectively. To account for van der Waals (vdW) interactions, the DFT-D3 method proposed by Grimme et al. [51] was employed in all calculations. The implicit solvent model implemented in the VASPsol software package (VASPsol 5.4.1) was used to treat the solvation effects [52,53]. To investigate the thermal stability of the DACs, ab initio molecular dynamics (AIMD) simulations [54] were performed for 10 ps at 500 K with a time step of 2 fs.

The Gibbs free energy change (Δ*G*) for each elementary step of the NRR was calculated using the computational hydrogen electrode (CHE) model proposed by Nørskov et al. [55]. The formula for calculating Δ*G* is as follows:

ΔG = ΔE + ΔE_ZPE_ ‒ TΔS + neU +ΔG_pH_

where Δ*E* is the reaction energy of each step calculated by DFT. Δ*E_ZPE_* and Δ*S* are the changes in zero-point energy and entropy at 298.15 K, respectively, obtained by calculating the vibrational frequencies. The vibrational frequencies and entropy of gas molecules (N_2_, H_2_, NH_3_) are obtained from the NIST database [56]. U represents the electrode potential, n represents the number of transferred electrons, and Δ*G_pH_* represents the free energy correction value at pH, defined as Δ*G_pH_ =* 2.303 × *k_B_T* × *pH*. In this work, the pH is set to 0. The highest positive Δ*G* value (Δ*G*_max_) throughout the process was employed to derive the limiting potential (*U_L_*), i.e., *U_L_* = −ΔG_max_/e.

## 4. Conclusions

This study systematically explored a range of double-atom catalysts, namely MM′@N_4_O_2_-G, for their potential as NRR electrocatalysts using density functional theory. Employing a multi-stage screening strategy, we identified eight candidate catalysts (MM′@N_4_O_2_-G, MM′ = MoFe, MoCo, MoCr, MoMn, MoRe, VFe, VMn, and VV) with both thermodynamic and electrochemical stability among 48 catalysts. The NRR mechanism was extensively studied for these catalysts. Computational results revealed that the NRR on VV@N_4_O_2_-G occurs through an enzymatic pathway, while the remaining seven catalysts follow a distal mechanism. Notably, in contrast to other systems, MoRe@N_4_O_2_-G facilitates nitrogen reduction by adsorbing two N_2_ molecules onto its surface, each anchored to a metal center. Further analysis of the electronic structures elucidated an “acceptance–feedback” mechanism between the active sites and N_2_ molecules. The limiting potentials for these eight catalysts ranged from −0.20 to −0.37 V. The volcano plot relationship between U_L_ and εd demonstrated the cooperative effect of two active sites in the DACs on the catalytic performance. Furthermore, we investigated the selectivity of the eight DACs and identified five potential NRR catalysts (MM′@N_4_O_2_-G, MM′ = MoFe, MoCo, MoCr, MoMn, and VV). MoCo@N_4_O_2_-G exhibits the most favorable catalytic performance among the five DACs, with a limiting potential of −0.20 V. AIMD simulations revealed the high thermal stability of these potential NRR catalysts at 500 K, suggesting feasibility for experimental synthesis and practical applications. We hope that this study will drive the exploration of the potential application of DACs in the NRR and other electrochemical reactions. 

## Data Availability

Data are contained within the article and Supplementary Materials.

## Supplementary Materials

The following supporting information can be downloaded at: https://www.mdpi.com/article/10.3390/molecules29040779/s1, Table S1: Number of electrons transferred from MM′ to the N_4_O_2_-G carrier (*Q* in e). Table S2: Comparison of Gibbs free energy changes for *N_2_ + N_2_(g) → *N_2_ + *N_2_ and *N_2_ + *N_2_ + H^+^ + e^−^ → *NNH + *N_2_ on seven heteronuclear DACs. Table S3: Comparison of Gibbs free energy changes for *N_2_ + H^+^ + e^−^ →*N_2_ + *H and *N_2_ + H^+^ + e^−^ → *NNH. Figure S1: Total energy variation of MM′@N_4_O_2_-G (MM′ = MoCo, MoMn, MoRe, MoCr, VV, VMn, and VFe) for 10 ps AIMD simulations at 500 K. Figure S2: Gibbs free energy diagrams of NRR on VFe@N_4_O_2_-G. Figure S3: Gibbs free energy diagrams of NRR on MoCr@N_4_O_2_-G, MoMn@N_4_O_2_-G, and VMn@N_4_O_2_-G. Figure S4: PDOS after N_2_ adsorption on MM′@N_4_O_2_-G (MM′ = MoFe, MoCr, MoRe, MoMn, VFe, VMn, and VV).
