# Supplementary material for: First-Principles Study of Bimetallic Pairs Embedded on Graphene Co-Doped with N and O for N2 Electroreduction
Source: Molecules. 2024 Feb 8;29(4):779. doi: 10.3390/molecules29040779 (PMC10891683; doi:10.3390/molecules29040779)
Supplement: Supplementary file 1 [file molecules-29-00779-s001.zip › molecules-2813237-supplementary.pdf]

Article

First-Principles Study of Bimetallic Pairs Embedded on Graphene Co-Doped with N and O for N_2 Electroreduction

Haozhe Dong, Hao Sun, Guanru Xing, Shize Liu, Xuemei Duan * and Jingyao Liu *

Institute of Theoretical Chemistry, College of Chemistry, Jilin University, Changchun 130023, China; donghz21@mails.jlu.edu.cn (H.D.); haosun22@mails.jlu.edu.cn (H.S.); xinggr21@mails.jlu.edu.cn (G.X.); szliu_0520@163.com (S.L.)

* Correspondence: duanxm@jlu.edu.cn (X.D.); ljy121@jlu.edu.cn (J.L.)

Table S1. Number of electrons transferred from MM' to the N_4O_2-G carrier (*Q* in e).

MM'	Q_M	$Q_{M'}$	MM'	Q_M	$Q_{M'}$	MM'	Q_M	$Q_{M'}$
MoTi	-1.09	-1.29	FeFe	-1.00	-1.06	VV	-1.22	-1.21
MoV	-1.12	-1.22	FeTi	-0.81	-1.44	VTi	-1.12	-1.40
MoCr	-1.19	-1.13	FeTa	-0.71	-1.54	VFe	-1.32	-0.80
MoMn	-1.13	-0.92	FeW	-0.84	-1.32	VW	-1.18	-1.30
MoFe	-1.22	-0.78	FeRe	-0.91	-1.17	VRe	-1.25	-0.95
MoCo	-1.31	-0.73	FeCr	-0.90	-1.28	VCr	-1.24	-1.12
MoNi	-1.54	-0.72	FeMn	-1.02	-1.22	VMn	-1.28	-1.09
MoCu	-1.35	-0.75	FeCo	-1.06	-0.86	VCo	-1.35	-0.75
MoMo	-1.13	-1.10	FeNi	-1.13	-0.83	VNi	-1.36	-0.75
MoW	-1.10	-1.29	FeCu	-1.07	-0.91	VCu	-1.38	-0.74
MoTa	-1.03	-1.55	MoRe	-1.20	-1.03	FeZn	-0.98	-1.23

Table S2. Comparison of Gibbs free energy changes for $*N_2 + N_2(g) \rightarrow *N_2 + *N_2$ and $*N_2 + *N_2 + H^+ + e^- \rightarrow *NNH + *N_2$ on seven heteronuclear DACs.

MM′@N$_4$O$_2$-G	ΔG ($*N_2+N_2(g) \rightarrow *N_2+*N_2$)	ΔG ($*N_2+*N_2+H^++e^- \rightarrow *NNH_{(Mo)}+*N_2$)	ΔG ($*N_2+*N_2+H^++e^- \rightarrow *NNH_{(M')}+*N_2$)	ΔG_{PDS}
MoFe	-0.34	0.32	1.18	0.24
MoRe	-0.68	0.35	1.76	0.56
MoCo	-0.41	0.32	0.62	0.20
MoCr	—	—	—	0.25
MoMn	—	—	—	0.27
VMn	—	—	—	0.23
VFe	—	—	—	0.44

Table S3. Comparison of Gibbs free energy changes for $*N_2 + H^+ + e^- \rightarrow *N_2 + *H$ and $*N_2 + H^+ + e^- \rightarrow *NNH$.

MM′@N_4O_2-G	ΔG ($*N_2+H^++e^-\rightarrow *N_2+*H$)	ΔG ($*N_2+H^++e^-\rightarrow *NNH$)
MoFe	0.32	0.24
MoRe	-0.09	0.56
MoCo	0.32	0.07
MoCr	0.27	0.25
MoMn	0.45	0.27

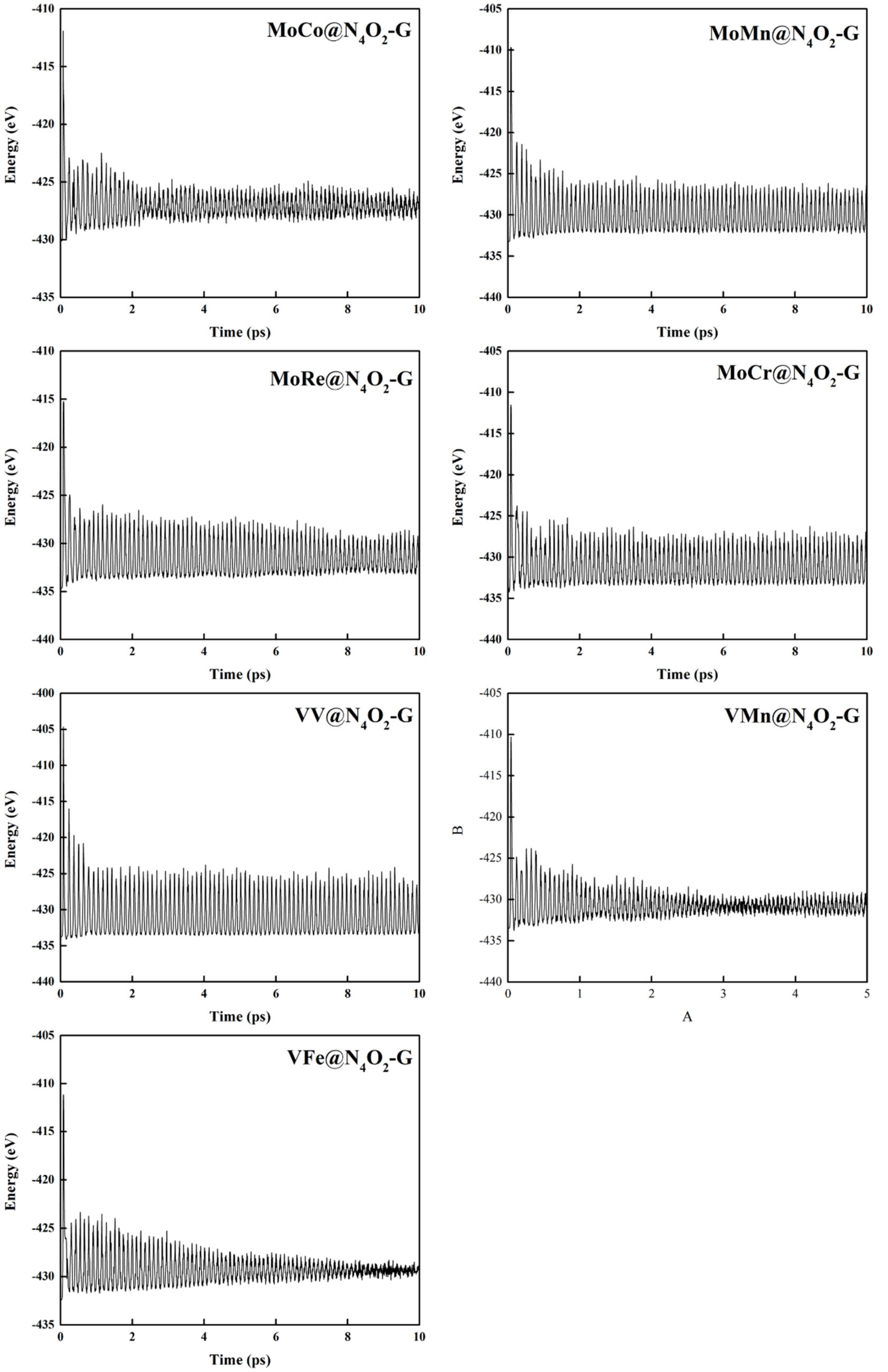

Figure S1. Total energy variation of MM′@N_4O_2-G (MM′ = MoCo, MoMn, MoRe, MoCr, VV, VMn and VFe) for 10 ps AIMD simulations at 500 K.

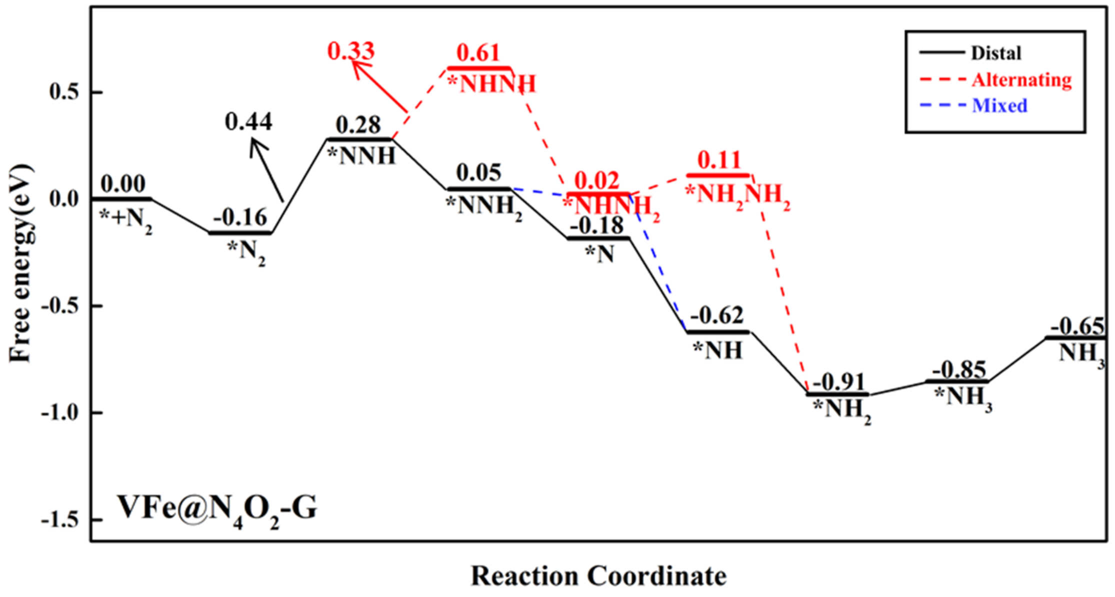

Figure S2. Gibbs free energy diagrams of NRR on VFe@N_4O_2-G.

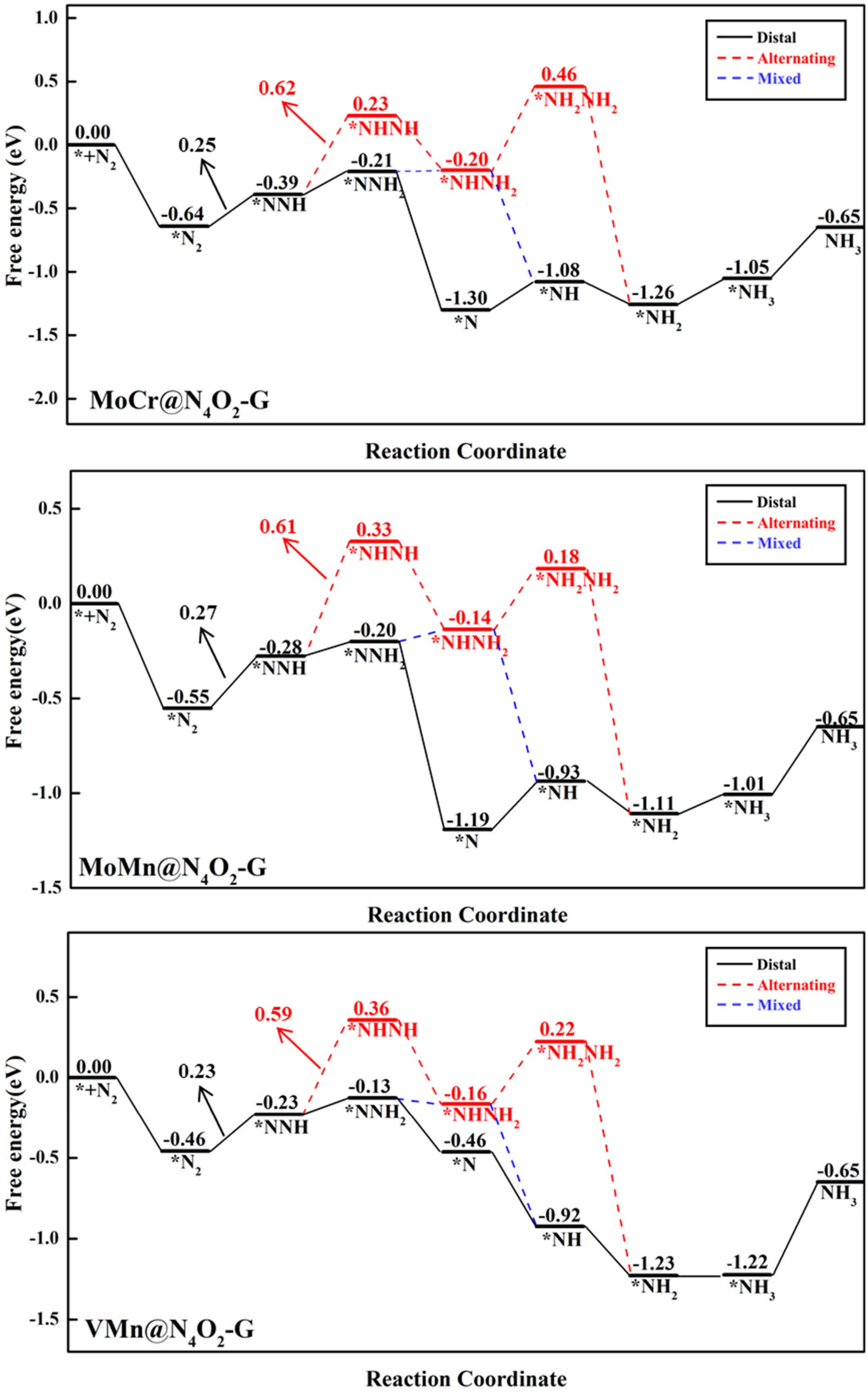

Figure S3. Gibbs free energy diagrams of NRR on MoCr@N_4O_2-G, MoMn@N_4O_2-G and VMn@N_4O_2-G.

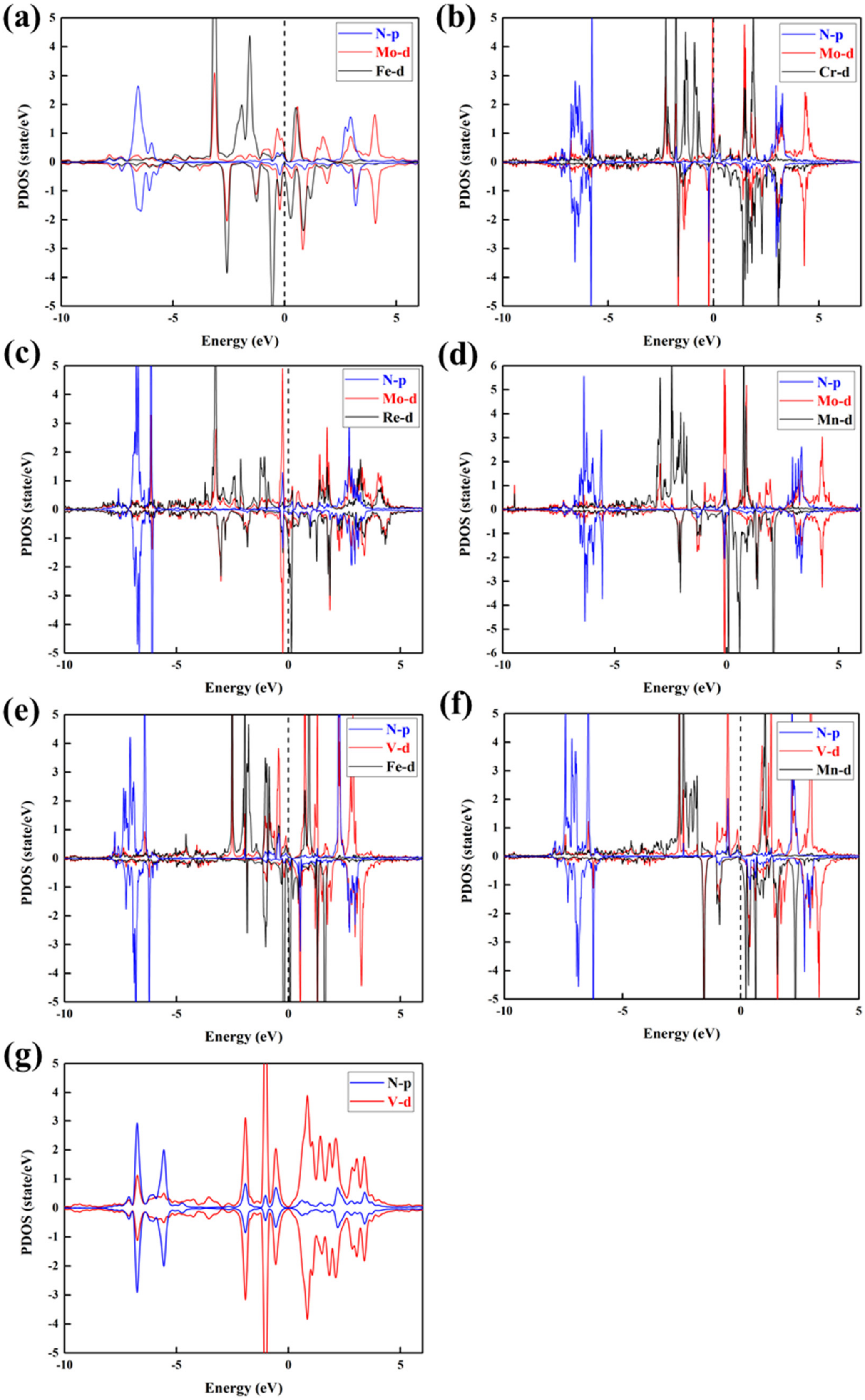

Figure S4. PDOS after N_2 adsorption on MM′@N_4O_2-G (MM′ = MoFe, MoCr, MoRe, MoMn, VFe, VMn and VV).
